# A Case of Nonunion Avulsion Fracture of the Anterior Tibial Eminence

**DOI:** 10.1155/2016/9648473

**Published:** 2016-03-29

**Authors:** Satoru Atsumi, Yuji Arai, Shuji Nakagawa, Hiroaki Inoue, Kazuya Ikoma, Hiroyoshi Fujiwara, Toshikazu Kubo

**Affiliations:** Department of Orthopedics, Graduate School of Medical Science, Kyoto Prefectural University of Medicine, 465 Kajiicho, Kawaramachi-Hirokoji, Kamigyo-ku, Kyoto 602-8566, Japan

## Abstract

Avulsion fracture of the anterior tibial eminence is an uncommon injury. If bone union does not occur, knee extension will be limited by impingement of the avulsed fragment and knee instability will be induced by dysfunction of the anterior cruciate ligament (ACL). This report describes a 55-year-old woman who experienced an avulsion fracture of the right anterior tibial eminence during recreational skiing. Sixteen months later, she presented at our hospital with limitation of right knee extension. Plain radiography showed nonunion of the avulsion fracture region, and arthroscopy showed that the avulsed fragment impinged the femoral intercondylar notch during knee extension. The anterior region of the bony fragment was debrided arthroscopically until the knee could be extended completely. There was no subsequent instability, and the patient was able to climb a mountain 6 months after surgery. These findings indicate that arthroscopic debridement of an avulsed fragment for nonunion of an avulsion fracture of the anterior tibial eminence is a minimally invasive and effective treatment for middle-aged and elderly patients with a low level of sports activity.

## 1. Introduction

Avulsion fracture of the anterior tibial eminence is an uncommon injury that usually occurs in children and adolescents at a frequency of about 3 in 100,000 [1]. Categorization of anterior tibial eminence fractures according to the Meyers and McKeever classification [2, 3] showed that most type I (nondisplaced) fractures are treated conservatively, whereas treatment methods of types II (partially displaced), III (completely displaced), and IV (comminuted) fractures have not been standardized. In the absence of bone union, knee extension will be limited by impingement of the avulsed fragment and knee instability will be induced by dysfunction of the anterior cruciate ligament (ACL). Knee instability may be treated by osteosynthesis [4, 5], one-stage ACL reconstruction [6], or debridement of the bony fragment [7, 8], with the choice of treatment based on each patient's age and level of sports activity, as well as the time after injury. This report describes a 55-year-old woman with a low sports activity level who experienced an avulsion fracture of the right anterior tibial eminence.

## 2. Case Presentation

A 55-year-old woman fell during recreational skiing and was diagnosed with Meyers and McKeever type II avulsion fracture of the right anterior tibial eminence. The fracture was initially treated conservatively. Sixteen months later, she visited our hospital for limited extension of the right knee joint. Physical examination showed a range of motion (ROM) of her right knee of 10°–140°; that is, she lacked 10° of extension. Lachman and pivot shift tests were negative.

**Figure 1:**
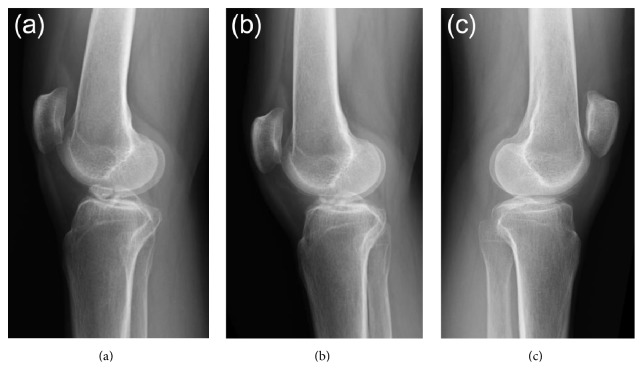
Plain radiography of our patient. (a) Lateral view of the right knee joint on first examination at our hospital, showing complete displacement of the avulsed fragment at the right anterior tibial eminence from the tibial bone mass and impingement of the fragment on the femoral intercondylar notch during knee extension. (b) Lateral view of the right knee joint 2 years after resection of the anterior region of the avulsed fragment at the anterior tibial eminence, showing no impingement of the avulsed fragment on the femoral intercondylar notch during knee extension. (c) Lateral view of the left knee joint at the time of initial examination at our hospital.

**Figure 2:**
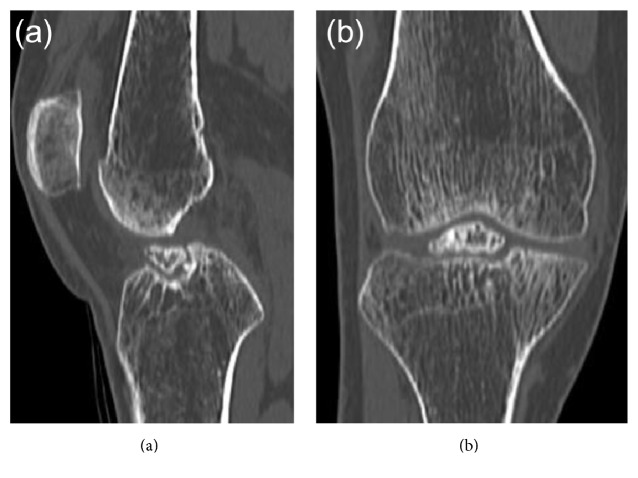
Preoperative computed tomography of our patient. (a) Sagittal and (b) coronal views, showing the avulsed fragment at the anterior tibial eminence of the right knee impinging on the femoral intercondylar notch.

**Figure 3:**
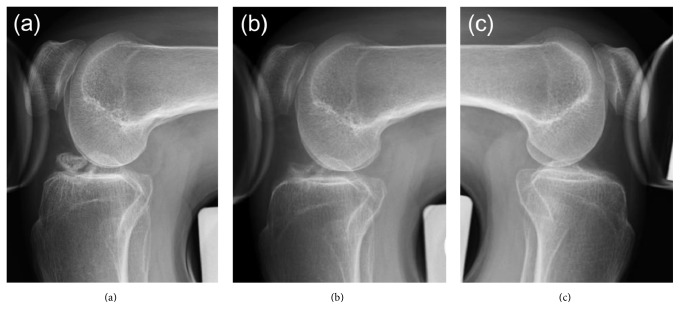
Stress radiographs with anterior drawer tests showing no difference between the (a, b) right and (c) left knees at (a, c) initial examination at our hospital and (b) 2 years after surgery.

**Figure 4:**
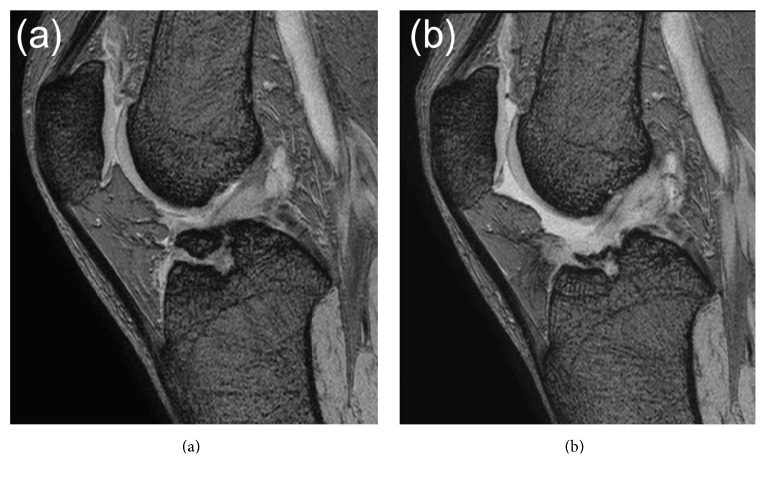
(a) Preoperative magnetic resonance imaging (MRI), showing no rupture in ACL fibers. (b) MRI 6 months after surgery showing only partial rupture of ACL fibers at the site of debridement.

Plain radiography and computed tomography (CT) showed the avulsed fragment at the ACL attachment site of the right tibia to be completely displaced from the tibial bone mass. This fragment impinged on the femoral intercondylar notch when the knee was extended (Figures 1(a) and 2). Stress radiographs during anterior drawer test showed no difference between the right and left knees (Figures 3(a) and 3(c)). Magnetic resonance imaging (MRI) showed that the ACL fibers were not ruptured (Figure 4(a)). The patient was diagnosed with nonunion after avulsion fracture of the right anterior tibial eminence.

**Figure 5:**
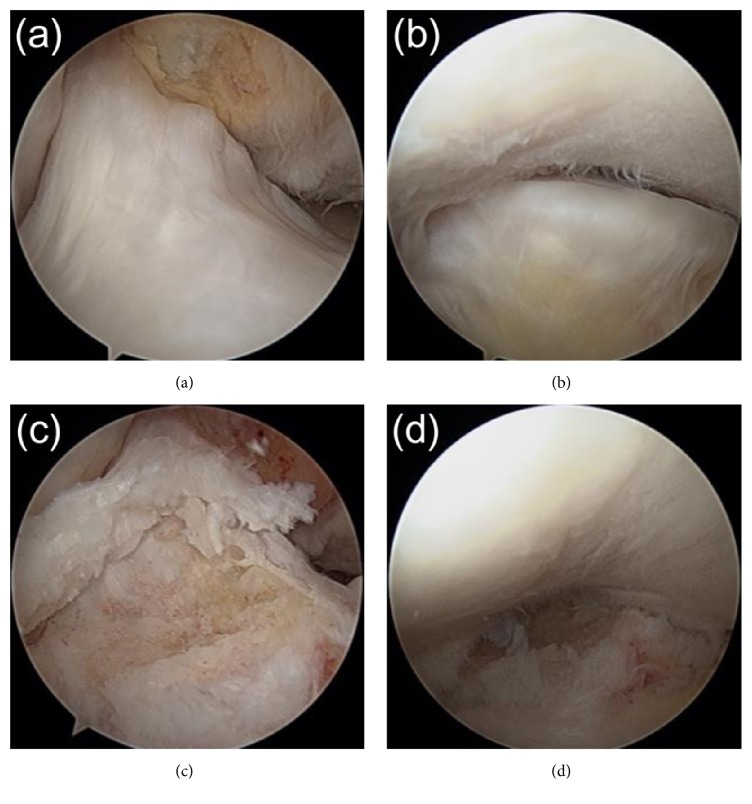
(a, b) Preoperative arthroscopic results, showing that (a) the parenchymal fibers of the anterior cruciate ligament (ACL) were normal, whereas (b) the avulsed fragment impinged on the femoral intercondylar notch as the knee was extended, limiting extension. (c, d) Arthroscopy after debridement, showing that (c) the anterior region of the avulsed fragment was shaved, the posterior region of the ACL attachment site of the tibia was conserved, and the tension of the ACL parenchyma was retained. (d) Excavation of the anterior region of the avulsed fragment resolved the limitation of knee extension.

Arthroscopy showed that the avulsed fragment at the anterior tibial eminence was dislocated upward, but there was no space between the bony fragment and tibial bone mass, and the fragment was surrounded by soft tissue. Examination of fragment stability using a probe showed mild looseness and fibrous union. The parenchymal fibers of the ACL were intact (Figure 5(a)). However, the avulsed fragment impinged on the femoral intercondylar notch as the knee was extended (Figure 5(b)), limiting extension. The anterior region of the avulsed fragment was shaved until the limitation of knee extension was resolved (Figure 5(d)). The posterior region of the ACL attachment site was conserved. A probe confirmed that tension of the ACL parenchyma was retained (Figure 5(c)).

Full weight bearing was permitted postoperatively. MRI 6 months after surgery showed only partial rupture at the site of debridement (Figure 4(b)), and the patient was able to climb a mountain at that time. Two years after surgery, there was no limitation of right knee extension (Figure 1(b)) or instability (Figure 3(b)).

## 3. Discussion

Surgical treatments of nonunion avulsion fractures of the anterior tibial eminence include osteosynthesis [4, 5], ACL reconstruction [6], and debridement of the bony fragment [7, 8]. Osteosynthesis was previously performed as open surgery but is now performed in a minimally invasive manner using arthroscopy. Arthroscopic osteosynthesis includes screw fixation using a cannulated cancellous screw [4], headless compression screw, or absorbable screw, with suture fixation of the bony fragment [5]. Osteosynthesis has been reported to be successful for nonunion avulsion fractures of the anterior tibial eminence. In a 7-year-old girl, however, this method required an above-the-knee cast for 6 weeks [4], and partial weight bearing was reported to be necessary until bone union [5]. The mean duration to union of nonunion avulsion fractures was 15 weeks [5].

Osteosynthesis alone may be insufficient to achieve knee stability if nonunion avulsion fracture of the anterior tibial eminence is accompanied by arthroscopically determined degeneration of the ACL fibers; rather, one-stage ACL reconstruction may be required [6]. It is important to consider age, sports activity level, time after injury, and concomitant injuries of the meniscus and joint cartilage in deciding whether to perform ACL reconstruction [9]. For example, conservative treatment of ACL injury in 52 patients aged 40–62 years resulted in a mean Lysholm and Gillquist score of 82 [10]. Manual tests continued to show joint instability, but these patients were able to participate in recreational sports activities and most were satisfied with the outcomes of nonsurgical treatment [10]. Although knee stability was found to be superior in 67 patients aged 40–59 years who underwent ACL reconstruction compared to that in 31 similarly aged patients who were treated conservatively, range of motion did not differ significantly [11]. Moreover, activity level returned to preoperative level in 67% of conservatively treated patients aged ≥50 years [11]. However, ACL reconstruction may result in limited range of knee motion [12]. As our patient was a 55-year-old woman with no subjective symptoms of knee instability, no local or imaging findings of ACL injury, and a low sports activity level, ACL reconstruction was not indicated.

Arthroscopy-assisted debridement of the avulsed fragment was shown to result in functional recovery in patients with nonunion after avulsion fracture of the anterior tibial eminence and a chief complaint of limited knee extension [7]. An algorithm indicated that arthroscopic debridement be performed when the range of motion was not improved by rehabilitation, whereas ACL reconstruction should be performed when knee joint instability remained after debridement [8]. Arthroscopic debridement in seven patients of mean age of 21.4 years resulted in the complete absence of knee joint instability, with all patients able to return to sports activities. Our patient underwent arthroscopic debridement because her chief compliant was limited extension. As arthroscopy showed an absence of parenchymal degeneration in the ACL fibers, only the anterior impinging region of the avulsed fragment was resected, while the posterior region of the tibial ACL attachment site was conserved. This treatment resolved the limitation of knee extension, and, in the two years after surgery, this patient has shown a favorable clinical course without knee instability. Findings in this patient suggest that arthroscopic debridement of the bony fragment is a minimally invasive and effective treatment for nonunion avulsion fracture of the anterior tibial eminence in middle-aged and elderly patients with a low level of sports activity.

## 4. Summary

A 55-year-old woman with nonunion after avulsion fracture of the anterior tibial eminence underwent arthroscopic debridement of the avulsed fragment. This treatment resolved her limited knee extension without inducing knee instability, and her subsequent clinical course was favorable. These findings indicate that arthroscopic debridement of an avulsed fragment is a minimally invasive and effective treatment for nonunion avulsion fracture of the anterior tibial eminence in middle-aged and elderly patients with a low level of sports activity.
